# Stochastic mRNA Synthesis in Mammalian Cells

**DOI:** 10.1371/journal.pbio.0040309

**Published:** 2006-09-12

**Authors:** Arjun Raj, Charles S Peskin, Daniel Tranchina, Diana Y Vargas, Sanjay Tyagi

**Affiliations:** 1 Courant Institute of Mathematical Sciences, New York University, New York, New York, United States of America; 2 Department of Molecular Genetics, Public Health Research Institute, Newark, New Jersey, United States of America; University of Geneva, Switzerland

## Abstract

Individual cells in genetically homogeneous populations have been found to express different numbers of molecules of specific proteins. We investigated the origins of these variations in mammalian cells by counting individual molecules of mRNA produced from a reporter gene that was stably integrated into the cell's genome. We found that there are massive variations in the number of mRNA molecules present in each cell. These variations occur because mRNAs are synthesized in short but intense bursts of transcription beginning when the gene transitions from an inactive to an active state and ending when they transition back to the inactive state. We show that these transitions are intrinsically random and not due to global, extrinsic factors such as the levels of transcriptional activators. Moreover, the gene activation causes burst-like expression of all genes within a wider genomic locus. We further found that bursts are also exhibited in the synthesis of natural genes. The bursts of mRNA expression can be buffered at the protein level by slow protein degradation rates. A stochastic model of gene activation and inactivation was developed to explain the statistical properties of the bursts. The model showed that increasing the level of transcription factors increases the average size of the bursts rather than their frequency. These results demonstrate that gene expression in mammalian cells is subject to large, intrinsically random fluctuations and raise questions about how cells are able to function in the face of such noise**.**

## Abstract 2

Counting individual mRNA molecules produced by a given gene provides evidence of random fluctuations in mammalian gene expression from cell to cell.

## Introduction

Many recent experiments show that genetically identical populations of bacteria and yeast can exhibit cell-to-cell variations in the amount of protein a gene produces [1–7]. These variations result in increased phenotypic diversity [8–14]. The variations are thought to arise from the typically small number of molecules involved in gene expression, with protein numbers often on the order of hundreds of molecules, mRNA on the order of tens of molecules, and the genes themselves often present in just one or two copies per cell. The factors leading to cell-to-cell variations can be classified as deriving from two sources: (a) variations in global, or extrinsic, factors, such as varying amounts of transcriptional activators, or (b) inherently random, or intrinsic, molecular events, such as the transcription of mRNA or translation of proteins [3,15].

While studies in bacteria have shown that variations have partially [3] or completely intrinsic [4] origins, studies in yeast suggested that variations arise mostly from extrinsic sources [1,5,16]. Recent studies with more accurate methods of analysis have, however, identified a more substantial intrinsic component to the variations in yeast [17,18]. In two of these studies [1,5], the authors developed models of gene expression postulating that the remaining intrinsic variability was due to random transitions of the gene itself between an active state, in which mRNA is transcribed at a high rate, and an inactive state, in which mRNA is transcribed at a much lower rate [19]. This theory predicts that the magnitude of variations in protein level (relative to the mean amount of protein) increases as the rate at which genes activate decreases. By experimentally varying the overall level of gene expression of fluorescent protein reporters, the authors obtained results consistent with this model. Other studies performed in higher eukaryotes by a variety of means, pioneered by the early work of Ko et al. [24], have indicated that significant cell-to-cell variations exist in these organisms as well [20–23].

However, direct detection of the proposed gene activation and inactivation events was not possible because new proteins from individual activation events were masked by proteins remaining from previous events as a result of the long half-lives of the fluorescent proteins used as the reporter. The use of fluorescent proteins is further limited by their low sensitivity: because individual molecules of fluorescent protein produce only small amounts of fluorescence, they are difficult to detect in the low numbers produced by many genes. This limitation is particularly troublesome in eukaryotic cells in general, and higher eukaryotes in particular, as the cellular volumes are much larger than those of bacteria, thus diluting the fluorescent protein concentration.

Given these limitations, the most direct way to detect gene activation and inactivation is to directly monitor the mRNA produced from the gene at the resolution of single molecules. Because the half-life of mRNA is typically much shorter than that of fluorescent proteins, their levels reflect more accurately the state of the gene. Moreover, by detecting single molecules, one would sidestep the issue of sensitivity. Furthermore, the presence of integral molecule counts would be especially valuable in precisely evaluating models of stochastic gene expression.

In this study, we explored cell-to-cell variation in gene expression in mammalian cells by accurately counting single molecules of mRNA through the use of fluorescence in situ hybridization (FISH). By obtaining precise measurements of these most immediate (and fast decaying) products of gene expression, we show direct evidence that genes transition infrequently between active and inactive states, resulting in large cell-to-cell variations in gene expression in clonal cell lines. In contrast to the mostly extrinsic variations observed in yeast, we show that these transitions are intrinsically random and not due to extrinsic factors and that they affect the expression of entire genomic loci. Furthermore, we found that the mRNA produced by the gene encoding the large subunit of RNA polymerase II is also produced in bursts, and that these bursts are uncorrelated with those from our reporter gene, indicating that the level of RNA polymerase II is not an important extrinsic determinant of cell-to-cell variations. We also analyzed the effect that the mRNA variations had on the proteins they encode and found that having a slow protein degradation rate can serve to buffer the mRNA variations. A mathematical model of gene activation and inactivation indicates that the mean number of mRNA produced per activation event (the “average burst size”) can be controlled by varying the amount of transcriptional activators in the cell.

## Results

### Detection of Individual mRNA Molecules

**Figure 1:**
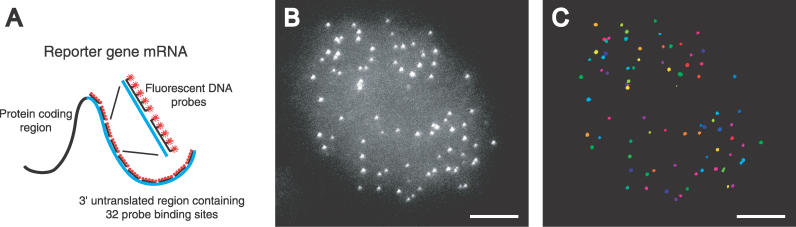
Detection and Quantification of Single Molecules of mRNA in Individual Cells (A) Schematic diagram depicting the mRNA detection method. Multiple fluorescent probes bind to each mRNA molecule, yielding a bright, localized signal. (B) Merged image of a three-dimensional stack of images from a CHO cell expressing the 7x-tetO gene depicted in (A), where each mRNA is hybridized to FISH probes that bind to the multimeric probe-binding sequence in its 3′-UTR (probe P1-TMR binding to the M1 multimer). Each spot corresponds to a single mRNA molecule. (C) Identification of the spots in the three-dimensional image stack in (C). Each particle found by the image-analysis algorithm is colored differently, showing that the algorithm is accurate and that individual molecules are uniquely identified. The scale bars are 5 μm long.

To directly observe random events of gene activation and inactivation, we measured cell-to-cell variations in the number of molecules of a specific mRNA. We accomplished this by integrating a reporter gene possessing a tandem array of probe binding sites into mammalian cells and utilized fluorescently labeled probes to visualize mRNA transcribed from the gene by FISH. To obtain single molecule sensitivity, we introduced 32 tandem copies of a 43–base-pair probe-binding sequence at the 3′ end of a coding sequence for a fluorescent protein (throughout this paper, we refer to this sequence array as M1). The construct was inserted into Chinese hamster ovary (CHO) cells by electroporation, and a stable cell line was isolated in which a single copy of the gene was integrated into the cells' genome. These cells were then fixed and subjected to hybridization with a single-stranded oligodeoxyribonucleotide probe that was both complementary to the tandemly repeated sequences and labeled with five well-dispersed fluorophore moieties (Figure 1A). The binding of so many fluorophores to each individual mRNA molecule resulted in signals so bright that each molecule was detectable as a diffraction-limited spot in a conventional wide-field fluorescence microscope (Figure 1B). To count the total number of mRNA molecules in each cell, optical slices spanning the full three-dimensional cellular volume were acquired (Video S1). The number of mRNA molecules in each cell was then measured using custom software to identify individual fluorescent spots in three dimensions from the image stacks (Figure 1C). We have shown previously that each spot corresponds to an individual mRNA molecule and that there is no significant loss of mRNA molecules during the FISH procedure [25], thus establishing that the method is a valid way to count the number of mRNA molecules in individual cells.

### Measurement of Cell-to-Cell Variations in Clonal Cells

**Figure 2:**
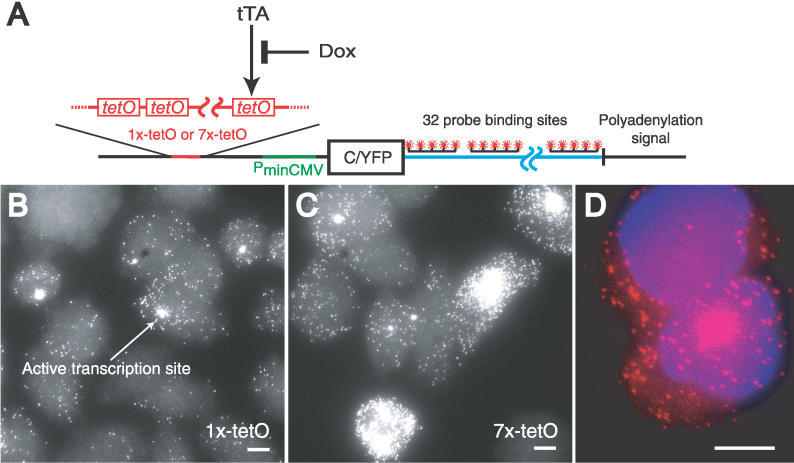
Cell-to-Cell Variation of mRNA Numbers in Clonal Cell Lines (A) Schematic diagram of the doxycycline-controllable promoters and the reporter genes that they control. Doxycycline binds to the tTA protein, thereby preventing it from binding to the *tet* operator. (B, C) Representative fields of cells from cell lines E-YFP-M1-1x and E-YFP-M1-7x, containing the 1x-tetO and 7x-tetO promoters, respectively, where each mRNA is hybridized to FISH probe P1-TMR and the image was obtained by merging a three-dimensional stack of images. (D) Two sister cells from cell line E-YFP-M1-7x displaying mRNA hybridized to FISH probe P1-TMR (red) and costained with DAPI (blue). The image represents one focal plane. The scale bars are 5 μm long.

To measure cell-to-cell variations in mRNA numbers in clonal cell lines, we generated stable CHO cell lines expressing our construct. The gene was placed under the control of a promoter whose expression could be controlled in mammalian cells (Figure 2A), and it was stably integrated into the genome via electroporation, resulting in the introduction of a single copy of the gene (as verified by Southern blotting; unpublished data). Observation of the mRNA synthesized in these cells showed that there were marked variations in the number of mRNA molecules from cell to cell (Figure 2B). The occasional larger bright areas that were observed are recently activated transcription sites [25,26] caused by a buildup of nascent mRNA that had not yet diffused away. The observation that these sites occur infrequently indicates that the mRNA is not being continually synthesized, but rather, it is synthesized during brief periods of time when the gene is transcriptionally active. We refer to these periods as transcriptional bursts. The rest of the time, the gene is in a transcriptionally inactive state, during which no mRNA molecules are synthesized and those synthesized earlier are degraded.

Quantitative evidence of the burst-like nature of transcription comes from comparing the number of mRNA in cells containing active transcription sites to those without active transcription sites. We found that of 97 randomly selected cells from cell line E-YFP-M1-7x (details of construct discussed below), the 23 containing transcriptional foci had an average of 244 mRNA per cell, as compared to 33 mRNA per cell in the 74 without any active transcription site (*p* < 10^−4^). Because the FISH method also gives the spatial location of the mRNA, we were also able to compare the relative numbers of mRNA in the nucleus and cytoplasm to study further the behavior of the transcriptional bursts. If transcription occurs in bursts, then one would expect to find more mRNA in the nucleus than in the cytoplasm when the gene is active, as the nuclear mRNA has not been exported. However, when the gene is in the inactive state, the nuclear mRNA will be exported without being replenished, resulting in a lower proportion of the total cellular mRNA being found in the nucleus. To examine such behavior, we costained the cells with DAPI after the hybridization and determined whether each mRNA was located in the cytoplasm or nucleus. Often, we found that cells without a transcriptional focus had only cytoplasmic mRNA, whereas cells with a transcription site usually had a large number of nuclear mRNA (Figure 2D). Statistically speaking, cells containing active transcription sites had a higher percentage of reporter mRNA in the nucleus (35%, 17 cells analyzed) than did cells without active transcription sites (25%, 22 cells analyzed) (*p* = 0.0093). Interestingly, the two cells depicted in Figure 2D are clearly descended from the same parent cell but seem to display different transcriptional behavior. This behavior is typical and indicates that variations global extrinsic factors such as position in the cell cycle are not the primary source of variation in the activity of the transgene; this is more systematically analyzed in the “Relative Contributions of Intrinsic and Extrinsic Factors to Variations in mRNA Level” section of the results.

Further evidence for transcriptional bursts comes from an analysis of the statistics of the distribution of mRNA molecules per cell over the entire cell population. If mRNA were produced at a constant rate, one would expect a Poisson distribution of mRNA per cell, in which case the mean number of mRNA molecules per cell and the variance (the square of the standard deviation) would be equal. However, we found that the mean was approximately 40 mRNA molecules per cell, while the variance was roughly 1,600 molecules squared, indicating that the mRNA is not synthesized at a constant rate, consistent with the occurrence of transcriptional bursts.

### Mechanisms Controlling Transcriptional Bursts

To investigate the mechanisms controlling transcriptional bursts, we altered the overall level of transcription both by changing the amount of transcriptional activator present in the cells and by altering the number of binding sites for that activator in the promoter. To accomplish this, the gene was inserted downstream from a minimal cytomegalovirus promoter, and either one or seven copies of the tetracycline-sensitive *tet* operator sequence were present upstream from the promoter (Figure 2A). Transcription from the promoter is only possible when a protein known as the tet-transactivator (tTA) binds to the operator sequence. tTA is a protein consisting of two domains: one that binds to the *tet* operator (derived from the TetR protein), and one that promotes transcription of nearby genes (the VP16 acidic activation domain). The tetracycline-like antibiotic doxycycline binds to the DNA-binding domain of tTA, preventing it from binding to DNA. By varying the level of doxycycline in the growth medium, we were able to control the level of free tTA in the cells [27].

**Figure 3:**
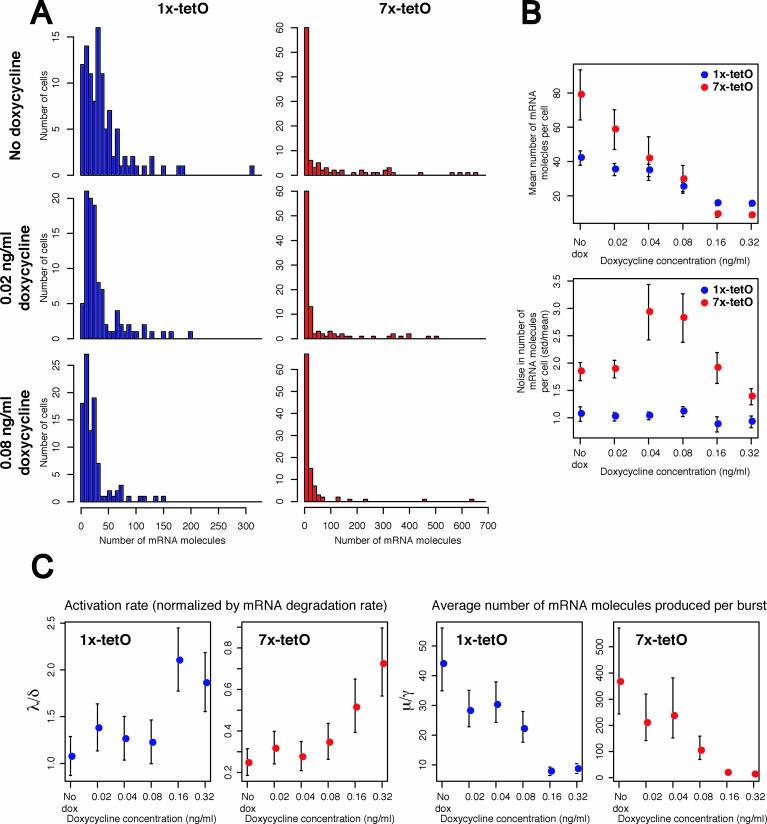
Statistical Analysis of Per-Cell mRNA Population Distributions (A) Histograms showing the distribution of mRNA molecules per cell for three doxycycline concentrations for both cell lines E-YFP-M1-1x (left) and E-YFP-M1-7x (right). (B) Graphs showing the population mean (top) and noise (defined as the standard deviation divided by the mean [bottom]) as a function of doxycycline concentration. Statistics were taken from the mRNA counts used in (A), and error bars were obtained by bootstrapping. (C) Activation rate (λ/δ) and average number of mRNA molecules produced per burst (μ/γ) for 1x-tetO (blue) and 7x-tetO (red) obtained from fitting the mRNA count data to the model of gene activation and inactivation by the maximum-likelihood method. Error bars reflect 95% confidence intervals.

Two constructs (1x-tetO and 7x-tetO) were stably integrated into CHO cells that had previously been modified to express tTA, resulting in the cell lines E-YFP-M1-1x and E-YFP-M1-7x, each containing a single copy of the respective reporter gene. Representative snapshots of clonal cell fields are shown in Figure 2B and 2C. We observed that the size of the bursts were larger in the E-YFP-M1-7x cell line than in the E-YFP-M1-1x cell line. We then varied the amount of doxycycline in the growth medium and measured the distribution of the number of mRNA molecules per cell across several fields of cells grown at each concentration of doxycycline (Figure 3A; mRNA counts given in Table S1). As expected, increasing the level of doxycycline resulted in a decrease in the mean number of mRNA molecules per cell (Figure 3B, top). However, we also found that the variability across the population (quantitatively measured by the “noise,” which we define as the standard deviation divided by the mean) remained constant over all doxycycline concentrations for the 1x-tetO construct but varied nonmonotonically for the 7x-tetO construct (Figure 3B, bottom). Moreover, we found that noise properties do not change if one considers mRNA concentration rather than absolute number (Figure S2, compare with Figure 3B, bottom). This is most likely because the primary source of variation is the activation state of the gene itself, which does not vary with the volume of the cell.

Both results are inconsistent with conventional stochastic models of gene expression [3,5,15,28] that predict that noise should increase steadily as the mean level of transcription decreases. To explain this behavior, we invoked a model of gene activation and inactivation [29] in which the gene undergoes infrequent transitions between a transcriptionally active state, during which many mRNA molecules are produced, and a transcriptionally inactive state, in which no mRNA molecules are produced (the model is analyzed in more detail in Protocol S1, where a complete formula for the mRNA distribution is presented). Using a fast numerical evaluation of the theoretical distributions resulting from this model, we were able to fit the experimentally obtained data to find expressions for the gene activation rate, λ (to within a factor of the mRNA degradation rate, δ), and the average number of mRNA produced during each burst, μ/γ (henceforth referred to as the average burst size) (Figure 3C). The mRNA half-life was determined by quantitative RT-PCR to be approximately 4 ± 1 h (Figure S1; see Materials and Methods for further discussion). The results of the fitting procedure show that either increasing the number of transcription factor binding sites or increasing the amount of intracellular transcription factors increases the average burst size. Based on our analysis, it is impossible to say whether this is due to a decrease in the rate of gene inactivation or an increase in the rate of transcription of the activated gene. This fact does, however, point to an important difference with the bacterial case, where gene activation and inactivation have typically been associated with transcription factor association and dissociation [4]. Were that the case, decreasing the amount of transcription factors would serve to decrease the rate of activation while leaving the rates of inactivation and transcription the same. In our data, the rate of gene activation appears to be fairly constant until the doxycycline concentration reaches a relatively high level, at which point it increases, arguing against the application of the bacterial model to our system. It is unclear why the rate of gene activation increases at the larger doxycycline concentrations, since decreasing the level of transcription factors should only decrease the rate of gene activation. This might be due to factors not included in our model, or some physical behavior on the part of the cell induced at higher doxycycline concentrations. However, our data generally indicate that modulating the concentration of transcriptional activators affects the overall level of transcription by altering the average burst size rather than its frequency.

### Relative Contributions of Intrinsic and Extrinsic Factors to Variations in mRNA Level

**Figure 4:**
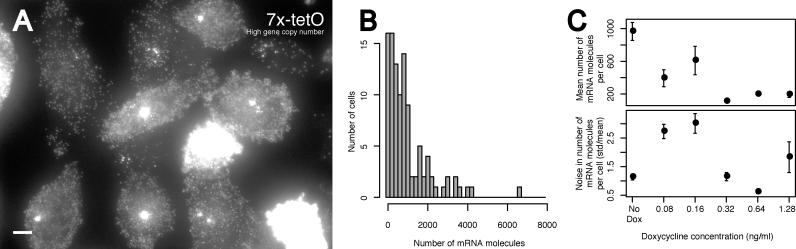
Cell-to-Cell Variations in mRNA Numbers in a Cell Line with Multiple Reporter Gene Integrations at the Same Gene Locus (A) Representative field from cell line L-GFP-M1-7x, generated by lipofection, where the mRNA was hybridized to FISH probe P1-TMR; the image was obtained by merging a three-dimensional stack of images. (B) Histogram showing the distribution of mRNA molecules per cell over for cell line L-GFP-M1-7x when grown in media containing no doxycycline. (C) Graphs showing the population mean (top) and noise (defined as the standard deviation divided by the mean [bottom]) as a function of doxycycline concentration. Error bars were obtained by bootstrapping.

If the variation in expression levels from one cell to another were truly due to random gene activation events, then the presence of multiple independently activating copies of the gene would result in less cell-to-cell variability in mRNA numbers (i.e., the noise should decrease). Intuitively, this can be seen by considering simultaneous coin tosses: if only one coin is tossed, it is either heads or tails, but if several coins are tossed at once, the chances of the set of them being close to 50% heads and 50% tails increases with the number of coins used. To test this possibility, we integrated multiple copies of our reporter gene into one region of the genome via cationic lipid-based transfection (lipofection), which simultaneously integrates tens to hundreds of gene copies, often in tandem and at the same locus, and isolated cell line L-GFP-M1-7x. Generally, the number of mRNA produced in this cell line was much larger than in E-YFP-M1-7x (with only one copy of the reporter gene), but the cell line still displayed massive cell-to-cell variations (Figure 4A) with a markedly skewed distribution (Figure 4B). Statistically, this is demonstrated by the fact that the noise characteristics of these two cell lines was similar; since the mean number of mRNA molecules per cell has increased roughly 10-fold (at no doxycycline) over the E-YFP-M1-7x cell line, one would expect the noise to decrease by a factor of √10 ≈ 3 if the genes expressed independently, but no such decrease was observed (Figure 4C; compare to Figure 3B).

There are two alternate explanations for this observation. One possibility is that the massive fluctuations seen in the number of mRNA molecules per cell are due to fluctuations in global factors that simultaneously affect the expression of all of the reporter genes (e.g., fluctuations in the levels of tTA or RNA polymerase II); this is usually referred to as extrinsic noise [3,15]. Alternatively, since the genes were integrated into the same genomic locus, it is possible that the genes express in a coordinated fashion in response to a random, local gene activation event (such as chromatin decondensation) that affects all nearby genes [1]. However, if local gene activation events occur at random, then genes located at distant sites would activate and deactivate independently. This type of noise, due to the random occurrence of events involved in gene expression, is usually referred to as intrinsic noise and is not dependent on global factors.

**Figure 5:**
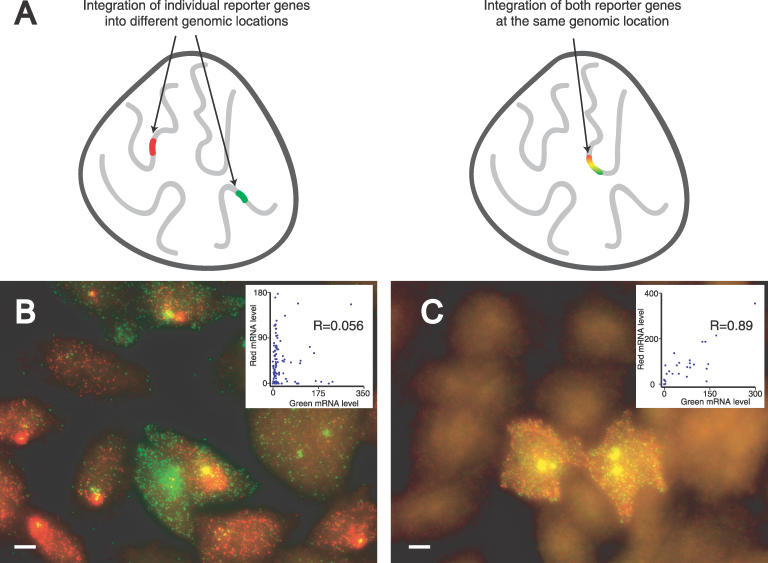
Dual-Reporter Experiments Showing Variations Are Intrinsically Random and Can Affect an Entire Gene Locus (A) Schematic depicting dual-reporter integration experiments, where the two reporters are either integrated in separate locations in the genome (left) or at the same locus (right). (B) Two-color overlay showing a merged three-dimensional stack of images of the cell line in which two different reporter genes (one expressing mRNA containing the M1 sequence array and the other expressing mRNA containing the M2 sequence array) are integrated into separate loci (cell line L-GFP-M1-7x+L-CFP-M2-7x); green corresponds to the signal from GFP-M1 mRNA and red corresponds to the signal from CFP-M2 mRNA (using probes P1-TMR and P2-Alexa-594, respectively). The relative mRNA levels of both genes were quantified by counting the mRNA of each color in a single optical slice in each cell (inset). (C) Two-color overlay showing a merged three-dimensional stack of images of the cell line in which the two distinct reporter genes are integrated into the same locus (cell line L-YFP-M1-CFP-M2), where the same FISH probes were used as in (B). The relative mRNA levels of both genes were quantified by counting the mRNA of each color in a single optical slice in each cell (inset). The scale bars are 5 μm long.

To explore these two alternative explanations, we constructed another reporter gene, CFP-M2, that encoded a cyan fluorescent protein and contained a different tandem array of probe-binding sequences in its 3′-UTR, denoted M2. This allowed its mRNA to be distinguished from the mRNA synthesized from reporter genes containing the M1 sequence array by performing FISH with an additional probe that binds to the M2 array but is conjugated to a differently colored fluorophore. In one series of experiments, this gene was integrated into a cell line (L-GFP-M1-7x) that already expressed a reporter gene containing the M1 array, resulting in the CFP-M2 reporter gene being integrated into a locus distant from the site of integration of GFP-M1 (Figure 5A, left). In a second series of experiments, the two reporter genes were integrated simultaneously via lipofection, resulting in both genes being integrated at the same locus (Figure 5A, right). If the variations in mRNA expression in each cell were due to global factors, bursts of mRNA synthesis from these distinct genes would likely occur simultaneously in the same cell regardless of their genomic location (i.e., both cell lines would show a strong correlation between the expression of both reporter mRNAs). However, if gene expression is controlled by local gene activation events affecting individual loci independently, the first cell line, in which the distinct reporter genes are integrated at different loci, would show no correlation in gene expression, but the second cell line, in which the distinct reporter genes are integrated at the same locus, would show a strong correlation in gene expression.

When integrated at separate loci (as evidenced by the presence of two distinct transcription sites), the two reporter mRNAs each individually displayed the large fluctuations observed previously (Figure 5B), yet the occurrence of those fluctuations were completely uncorrelated with each other (*R* = 0.056, *p* = 0.57) (Figure 5B, inset). However, when the two genes were integrated at the same locus (as evidenced by a single, dual-colored transcription site; Video S2), the genes produced both types of mRNA in simultaneous bursts (Figure 5C and inset; *R* = 0.89, *p* = 1.2 × 10^−38^). Taken together, the results of these experiments show that infrequent gene activation and inactivation events control the variability in mRNA levels, and these events occur randomly and are not dependent on global, extrinsic factors. Moreover, the results imply that these gene activation events are spatially extended, in that they affect whole regions of the genome at once.

### Cell-to-Cell Variations in the mRNA Encoding the Large Subunit of RNA Polymerase II

**Figure 6:**
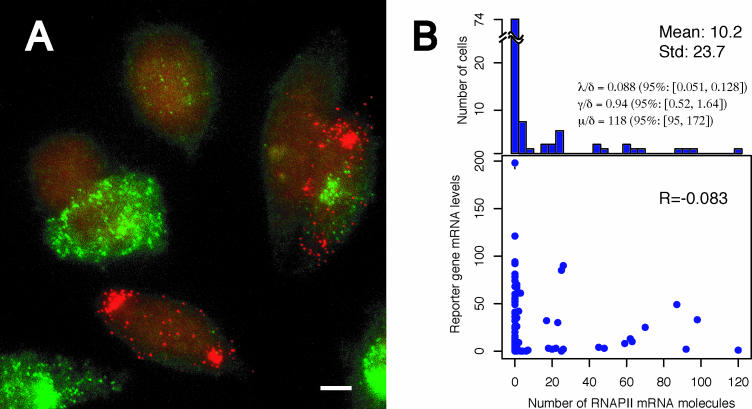
Cell-to-Cell Variations in the mRNA Encoding the Large Subunit of RNA Polymerase II (A) Representative field of cell line E-YFP-M1-7x upon performing FISH with differently colored probes for both YFP-M1 mRNA and the mRNA encoding the large subunit of RNA polymerase II. The image is a two-color overlay, where green corresponds to the signal from YFP-M1 mRNA (one optical slice) and red corresponds to the signal from RNA polymerase mRNA (merged three-dimensional image stack). The probes used were P1-Cy5.5 and P3-TMR. (B) Histogram showing the distribution of RNA polymerase mRNA molecules per cell (top) and scatterplot (bottom) showing reporter mRNA levels (quantified by counting the mRNA in a single optical slice) and RNA polymerase mRNA levels (quantified by counting all mRNA) in each cell. Gene activation (λ/δ), inactivation (γ/δ), and transcription rates (μ/δ) (normalized by the mRNA decay rate δ) are given with 95% confidence intervals as indicated. The scale bar is 5 μm long.

To further examine the role of global, extrinsic factors, we checked for fluctuations in a putative extrinsic factor, RNA polymerase II, to see if the level of expression of its mRNA correlated with the level of expression of the mRNA from a reporter gene. We were able to image individual molecules of the natural mRNA encoding the large subunit of RNA polymerase II by exploiting the presence of a naturally occurring 21-nucleotide-long sequence that is repeated 52 times in the mRNA. We used a FISH probe for the repeated sequence that was labeled with a distinctively colored fluorophore, and we counted fluorescent spots similar to those observed previously (Figure 6A). We found that this mRNA also displayed bursts of mRNA synthesis and that its distribution across a field of cells was similar to those observed for the reporter genes, with a variance over 50 times the mean (Figure 6B, top). To check for a correlation between the level of this mRNA and that of a reporter gene (in cell line E-YFP-M1-7x), we also quantified the level of mRNA expression of the reporter gene in the same cells. No significant correlation was found (*R* = 0.083, *p* = 0.41) (Figure 6B, bottom). By using the model of gene activation and inactivation, we were able to estimate the rates of gene activation, inactivation, and transcription to within a factor of the mRNA half-life (see Figure 6B for parameter values and confidence intervals), indicating that the activation was indeed infrequent and burst-like. These results show two things: (a) synthesis of the mRNA from natural genes can also be burst-like and (b) fluctuations in the number of mRNA molecules encoding the large subunit of RNA polymerase II are not a source of noise in the expression of other genes.

### Propagation of mRNA Variability to Protein Levels

To investigate the effects that burst-like transcription of mRNA had upon intracellular protein levels, we simultaneously quantified the number of mRNA and the fluorescent proteins they encoded in individual cells. To assess the effects that the rate of protein degradation had upon protein variability, we performed this analysis on a cell line expressing a fluorescent protein that was actively degraded and another cell line expressing a fluorescent protein with no active degradation.

**Figure 7:**
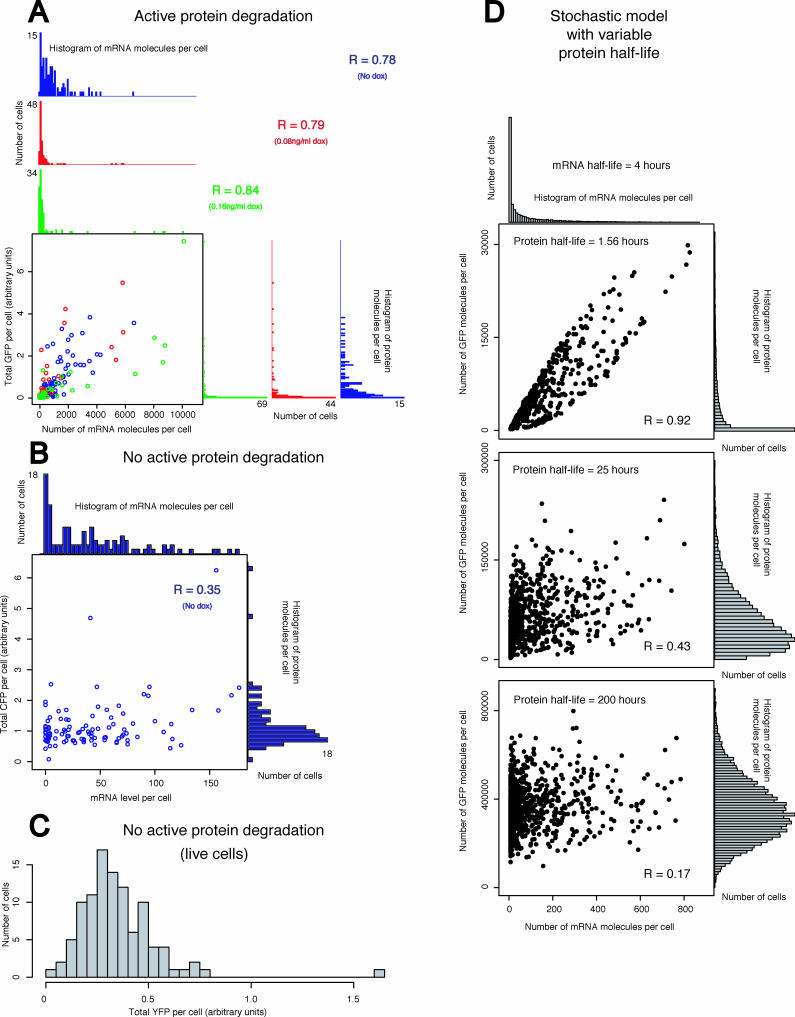
Propagation of mRNA Variations to Variations in Protein Levels (A) Scatterplot of total GFP and mRNA numbers in individual cells from cell line L-GFP-M1-7x grown under conditions of no doxycycline (blue), 0.08 ng/ml doxycycline (red), and 0.16 ng/ml doxycycline (green). Marginal histograms indicate the distribution of reporter mRNA per cell (top) or total GFP (right) for all the growth conditions. (B) Scatterplot of total CFP and mRNA levels (obtained from a single optical slice) in individual cells from cell line L-GFP-M1-7x+L-CFP-M2-7x grown under conditions of no doxycycline. Marginal histograms indicate the distribution of reporter mRNA per cell (top) or total GFP (right) for all the growth conditions. (C) Histogram of total YFP per cell from cell line E-YFP-M1-7x. YFP was quantified in live cells to minimize loss of fluorescence due to fixation and permeabilization. (D) Scatterplots and associated marginal histograms showing the results of stochastic simulations of the model of mRNA and protein dynamics presented in Protocol S1. The mRNA dynamics were the same for all cases, but the protein degradation rate was increased as indicated. Details of the simulation procedures and exact values of the parameters used are given in the Materials and Methods section.

For the case of active degradation, we used a cell line stably expressing the GFP-M1 reporter gene, which encoded a green fluorescent protein (GFP) that had been tagged at the C-terminus with a short amino acid sequence rich in proline, glutamic acid, serine, and threonine (d2EGFP) that targets the protein for active degradation (with a half-life of approximately 2 h) and examined the correlations between the mRNA and protein levels (Figure 7A). We found that the levels correlated quite well, with correlation coefficients larger than 0.78 (*p* < 2.6 × 10^−22^) over a range of transcriptional strengths. Moreover, the distribution of total protein levels appeared rather similar to those of the mRNA (Figure 7A, marginal histograms on top, mRNA, and right, protein).

For the case of no active protein degradation, we used another cell line, this time expressing the CFP-M2 reporter gene, in which the fluorescent protein did not contain any degradation tags. We found that the correlation between the mRNA and protein levels was significantly lower than before (*R* = 0.35, *p* = 1.9 × 10^−4^) (Figure 7B). Moreover, while the mRNA distribution was still heavily skewed with long tails, the protein distribution was somewhat less skewed. We also examined the distribution of proteins in live cells from cell line E-YFP-M1-7x, whose reporter gene also encodes a fluorescent protein that is not actively degraded. In this case, the single-copy integration produced too few proteins for us to detect after the fixation procedure, preventing us from simultaneously measuring the mRNA levels, but we found that the fluorescent protein levels in live cells also displayed a much less skewed distribution as compared with the actively degraded proteins (Figure 7C).

To explain the differences in protein distributions and correlation between the actively degraded and nondegraded cases, we added protein dynamics to the model of mRNA dynamics and examined the behavior of this model through the use of Gillespie's stochastic simulation algorithm [30]. (The parameters used for the simulations were those obtained from cell line E-YFP-M1-7x under conditions of no doxycycline, although the qualitative features observed do not depend heavily on the specific parameters used.) These simulations show that decreasing the rate of protein degradation results in a sharp decrease in the correlation between the mRNA and protein levels (Figure 7D). Also, the protein distribution changed from being heavily skewed to being more Gaussian in nature (Figure 7D, right marginal histograms), even though the mRNA distribution remained heavily skewed in all cases (Figure 7D, top marginal histogram). Intuitively, this is because proteins with fast protein degradation rates will be abundant only when the mRNA encoding it is abundant, resulting in a high correlation and similar distributions. However, if the proteins degrade very slowly, then proteins from earlier transcriptional bursts may still be present when new bursts occur. In this case, the transcriptional bursts merely serve to occasionally “top up” the amount of fluorescent proteins from time to time, resulting in less skewed distributions and a lower correlation between mRNA and protein numbers. Qualitatively, these predictions correspond well with our experimental observations.

## Discussion

We have shown that the mRNA levels of both reporter genes and native genes display large cell-to-cell variations in mammalian cells due to intrinsically random, infrequent events of gene activation. These burst-like fluctuations are not restricted to engineered reporter genes, but occur in natural genes as well, as demonstrated for the mRNA encoding the large subunit of RNA polymerase II. We have further shown that these events are controlled by gene regulatory mechanisms, such as the level of activator proteins and the number of transcription factor binding sites, and can affect regions of the genome rather than just specific genes.

Moreover, we have found that the variations are intrinsically random, rather than due to global extrinsic factors. This contrasts with the results of previous studies in lower eukaryotes [1,5,16], although some qualification of those studies may be required, as some extrinsic effects may simply be related to fluctuations in cell volume (see Protocol S1 for a further comparison to previous studies). This finding is significant, because extrinsic variations are often due to fluctuations in transcription factors [1,3] and cell cycle [16], which means they are at least partially regulated, whereas intrinsic variations are by definition uncontrollable.

We have also shown that the statistics of these variations are well described by a model in which the only sources of randomness are random events of gene activation and inactivation, implying that one can safely ignore the randomness inherent in the chemical reactions describing transcription and translation. This is qualitatively different from the bacterial case, where such reactions are thought to be the dominant source of variability in gene expression [3,7,28,31–33], despite some recent evidence of relatively mild burst-like behavior in Escherichia coli [4]. Other studies performed in higher eukaryotes have found similar behavior to that we have observed, albeit by different means [20–22,24]. In particular, the work of Chubb et al. [23] showed through temporal measurements of active transcription sites that genes do indeed undergo random transitions between transcriptionally active and inactive states, providing a powerful corroboration of our model. Their study used the MS2 method of mRNA detection, which has previously been used to monitor real-time kinetics of gene activity [26].

### Possible Physical Mechanisms for Transcriptional Bursting

The most likely sources for the transcriptional bursts are random events of chromatin remodeling [1,5]. If this is the case, then gene activation would correspond to chromatin decondensation and gene inactivation would correspond to chromatin condensation, facilitated by the activity of histone acetyltransferases and deacetyltransferases, respectively. Our experiments with two reporter genes integrated in tandem or at different locations of the genome support this idea (Figure 5). When the genes are located in distant regions of the genome, they burst independently, but when they are located near each other, they burst together. This is consistent with previous studies in which VP16-mediated decondensation was observed to extend over a region much larger than a single gene [34,35].

If the decondensation of chromatin is a prerequisite for gene activation, then the nucleation of this decondensation will be a significant rate-limiting step. The structure of chromatin at the level of nucleosome stacking suggests that the “breathing” events that permit the entry of transcriptional regulators will be infrequent [36]. However, once a transcription regulator is able to bind to its target site on the DNA exposed during a breathing event, it would attract histone acetyltransferases and thereby keep the immediate context of chromatin accessible. Of course, the rate of the nucleation will likely depend on the actual location of the genome in question, with some areas exhibiting lower nucleation frequencies than others.

The fact the mRNA is produced in bursts points to new means by which the cell may control transcription. There are three apparent means by which a cell would be able to upregulate a gene's transcription: it could (i) increase the rate of gene activation, (ii) increase the rate of transcription when the gene is in the active state, or (iii) decrease the rate of gene inactivation (the opposite behaviors, of course, apply should a cell decide to downregulate a gene's transcription). These mechanisms, while all resulting in the same *average* increase in transcription, differ markedly in the nature of the cell-to-cell variations induced. Our data indicate that in our system, either case (ii) or (iii) applies, whereas case (i) does not; in other words, the average burst size is being modulated rather than their frequency. The observation that altering the level of transcriptional activator does not reduce the rate of gene activation supports this hypothesis. Furthermore, the fact that altering the level of transcriptional activator does not reduce the rate of gene activation again argues for the intrinsic nature of the variations observed: if the primary source of cell-to-cell variation is the infrequent events of gene activation and those events are independent of the level of transcriptional activator, then the variations are likely due to some intrinsic fluctuations gene activation that do not depend on transcriptional activators. If gene activation does indeed correspond to chromatin remodeling, this points to the possibility that the nucleation of chromatin decondensation at a gene locus may be an inherently random event that does not require the presence of transcription factors but, once initiated, requires those factors to sustain the decondensed state.

### Mathematical Model

Our mathematical treatment of stochastic gene expression is rather different than methods based on moment generating functions [28] and applications of the fluctuation-dissipation theorem [31] in that we are generally more concerned with obtaining some information about the nature of the entire distribution rather than simply finding formulas for the first two moments (although we do provide alternate derivations of such formulas in Protocol S1). While moment computations are very useful in evaluating stochastic models in bacteria, we believe that information regarding the entire distribution is critical to understanding the observed burst-like events that resulted in heavily skewed distributions, since such distributions are not very well described by population means and variances. Our use of exact solutions for the complete distribution enabled us to perform rigorous statistical determinations of key model parameters. Of course, obtaining expressions for such distributions is generally difficult for most chemical master equations, and so we anticipate that the use of telegraph-like signals (as elucidated in Protocol S1) may lead to significant simplifications. The primary assumption that allows the use of such models is that the randomness associated with individual events of transcription and translation is relatively mild compared with that arising from random gene activation and inactivation. We anticipate this assumption to be generally valid in higher eukaryotes, especially given the role that chromatin dynamics plays in their expression patterns. Such methods may find particular utility in the study of the dynamics of cell signaling networks, which have been shown to exhibit the burst-like variations observed here [24,37].

### Implications for Cellular Function

In a wider sense, the presence of such large, unpredictable fluctuations in gene expression may initially appear to be a significant impediment to the functioning of a cell. In particular, given its essential role in cellular function, it is surprising that the gene encoding the large subunit of RNA polymerase II also displays fluctuations on the order of those seen in the reporter genes. Our analysis of protein levels yields a resolution to this apparent paradox: if the degradation rate of the proteins is sufficiently small, then the variations in protein level will be buffered because the proteins from new bursts serve only to “top up” the proteins already present from previous bursts. This suggests that essential genes whose mRNA expression is burst-like should have relatively stable proteins. Moreover, there are other manners in which protein variations may be further reduced. For instance, should two different proteins, each bursting independently, form a heteromeric complex, then the variations in the number of complexes will be somewhat buffered from the variations in each component.

Conversely, there may also be situations in which burst-like expression of unstable proteins may be desirable as well. Many examples of such situations exist in bacteria and yeast, often as a result of multistable behavior [8,10–12,14,38]. However, while such phenotypic variability may be advantageous for unicellular organisms because each cell is essentially identical, the same reasoning does not necessarily apply to multicellular organisms, in which the diversity of cellular function is controlled by the organism's developmental program. It is possible, though, that higher eukaryotes might also be able to exploit this variation to achieve a multitude of cellular behaviors in otherwise homogeneous tissues and cell types, leading to, for example, mosaic phenotypes [39] or transitions between phases in the viral life cycle [12]. In multicellular organisms, however, the reasoning behind the need for phenotypic variability is somewhat different than in unicellular organisms, since the variability is not designed to take advantage of an unpredictable environment but rather to achieve varied function or behavior within a relatively constant environment. We expect that distinctions such as these will result in interesting differences in the properties of stochastic gene expression in unicellular and multicellular organisms.

## Materials and Methods

### Multimer construction.

Construction of the DNA fragment with 32 probe binding sites in the pGEM-f11(z-) cloning vector (Invitrogen, Carlsbad, California, United States) was performed by the method described in Robinett et al. [40]. The mutually complementary oligonucleotides used to produce the M1 32-mer are M1-forward: TCGACCGATCGTGGCCTAAGGAGTTTATATGGAAACCCTTACCAGCCGCTCGAGCCGAGG and M1-reverse: GATCCCTCGGCTCGAGCGGCTGGTAAGGGTTTCCATATAAACTCCTTAGGCCACGATCGG.

The underlined portions of the sequence correspond to the SalI, XhoI, and BamHI sites used for integration into the host vector. A similar procedure was used to produce the M2 32-mer described by Vargas et al. [25]. The following oligonucleotide was used: M2-forward: TCGACCAGGAGTTGTGTTTGTGGACGAAGAGCACCAGCCAGCTGATCGACCTCGAGCCGAGG, along with the corresponding reverse oligonucleotide designed in the same manner as M1-reverse. The resulting plasmids were pGEM-M1-32x and pGEM-M2-32x.

### Creation of the reporter genes.

The reporter genes were constructed by adding an open reading frame for yellow fluorescent protein (YFP) and cyan fluorescent protein (CFP) upstream of the M1 and M2 multimers in the pGEM-M1-32x and pGEM-M2-32x plasmids, respectively. The sequences encoding YFP and CFP were amplified via PCR from pDH3 and pDH5 (University of Washington, Yeast Resource Center, Seattle, Washington, United States) and inserted in front of the M1 and M2 multimers between the SacI and SalI restriction sites, also introducing a BglII restriction site between the SacI site and the start codon of the open reading frame.

### Integration into expression vectors.

These reporter genes were then integrated into expression vectors enabling their expression in mammalian cells. The base vectors chosen were the pTRE2Hyg, pTRE2Pur, and pTRE-d2EGFP vectors (Clontech, Palo Alto, California, United States). Each contains a tetracycline-responsive promoter containing seven copies of the tet operator followed by a minimal cytomegalovirus promoter and a polyadenylation signal. Additionally, the pTRE2Hyg and pTRE2pur vectors enabled selection by appropriate quantities of hygromycin B (Invitrogen) or puromycin (Sigma, St. Louis, Missouri, United States).

To create a vector with one copy of the tet operator, we amplified the promoter region of pTRE2Hyg using a primer containing one copy of the tet operator. This was then cloned back into the pTRE2Hyg promoter site, replacing the native promoter and creating the plasmid pTRE2Hyg1x. The YFP-M1 construct was then extracted from the pGEM host vector with BglII and NotI and then inserted into the pTRE2Hyg and pTRE2Hyg1x vectors between the BamHI and NotI sites. The CFP-M2 construct was similarly inserted into the pTRE2Hyg and pTRE2Pur vectors. This created the plasmids pTRE2Hyg-YFP-M1, pTRE2Hyg1x-YFP-M1, pTRE2Hyg-CFP-M2, and pTRE2Pur-CFP-M2. The M1 multimer was also inserted into 3′-UTR in the pTRE-d2EGFP vector between the BamHI and EcoRI sites, creating the plasmid pTRE-d2EGFP-M1. All constructs were verified by sequencing.

### Creation of cell lines.

All cell lines were derived from the CHO-AA8-Tet-off cell line (Clontech), which possesses a stably integrated gene expressing the tetracycline-controlled Tet-off transactivator. Cell lines E-YFP-M1-1x and E-YFP-M1-7x, containing the 1x-tetO and 7x-tetO constructs, were generated by electroporation (Bio-Rad, Hercules, California, United States) using plasmids pTRE2Hyg1x-YFP-M1 and pTRE2Hyg-YFP-M1, respectively. The electroporator settings were 360 V and 975 μF using 10 μg of plasmid DNA linearized with XmnI added to 10^7^ cells in 500 μl of PBS in a 4-mm gap cuvette. The multiple-copy integration clones were generated using LipofectAMINE 2000 (Invitrogen) by following the manufacturers instructions. Cell line L-GFP-M1-7x was created by transfecting the CHO-AA8-Tet-off cell line with the pTRE-d2EGFP-M1 plasmid linearized with ScaI. To create the cell lines in which two reporter genes were integrated in different loci, cell line L-GFP-M1-7x was transfected using LipofectAMINE 2000 with the plasmid pTRE2Hyg-CFP-M2, linearized with XmnI; the resultant cell line is L-GFP-M1-7x+L-CFP-M2-7x. To create the cell line in which two reporter genes were integrated in the same locus, the CHO-AA8-Tet-off cell line was simultaneously cotransfected with equal amounts of pTRE2Hyg-YFP-M1 and pTRE2Pur-CFP-M2, resulting in cell line L-YFP-M1-CFP-M2. Cell lines were isolated after transfection by either electroporation or lipofection via selection with the appropriate antibiotic (hygromycin B or puromycin) and then purified by serial dilution. That only one copy of the transgene was integrated into cell lines E-YFP-M1-1x and E-YFP-M1-7x was verified by Southern blotting upon digestion of genomic DNA with the restriction enzyme BglII. Several cell lines were isolated following transfection; all exhibited similar phenotypes to the cell lines analyzed in this paper. Cell lines obtained from lipofection with pTRE-d2EGFP-M1 were isolated not by antibiotic selection but instead by directly identifying fluorescent cell clusters and purifying by serial dilution. Stability of the gene was verified by DNA FISH (unpublished data).

### Cell culture.

Cells were cultured in the alpha modification of Eagle's minimum essential medium (Sigma) supplemented with 10% TET-System-Approved fetal bovine serum (Clontech). The growth medium was supplemented with a low concentration of the selective antibiotic to ensure stability of the transfected gene. Appropriate amounts of doxycycline were added as indicated, and cells were grown at the desired concentration of doxycycline for 4 d to minimize any transient effects. The doxycycline concentration experiments were all performed in parallel with the same batch of media to minimize differences due to serum composition, etc.

### Construction of probes for in situ hybridization.

The probes used for the in situ hybridization were DNA oligonucleotides synthesized on an Applied Biosystems (Foster City, California, United States) 394 DNA synthesizer using mild phosphoramidites (Glen Research, Sterling, Virginia, United States). The oligonucleotide sequences were P1: 5′-CGGCRGGTAAGGGRTTCCATARAAACTCCTRAGGCCACGA-3′; P2: 5′-RCGAGGTCGARCAGCTGGCTGGRGCTCTTCGRCCACAAACA-3′; and P3: 5′-AGAGGRGGGCGAGRAGCRGGGAGAGGRGGGCGAGRAGCRGGG-3′, where P1 and P2 are complementary to the repeat sequences in M1 and M2, respectively, and P3 is complementary to a consensus sequence for the 52 repeats in the RNAPII subunit A cDNA sequence. The “R”s represent locations where an amino-dT was introduced in place of a regular dT. The oligonucleotides were synthesized on a controlled pore glass column (Glen Research) that introduced an additional amine group at the 3′ end of the oligonucleotide. The probes' amine groups were then coupled to the fluorophores Cy5.5, Alexa 594, and tetramethylrhodamine (Molecular Probes, Eugene, Oregon, United States) to create the following probes: P1-TMR, P1-Cy5.5, P2-Alexa-594, and P3-TMR. The probes were purified on an HPLC column to isolate oligonucleotides displaying the highest degree of coupling of the fluorophore to the amine groups.

### In situ hybridization.

Cells were cultured in multichambered coverglass (Lab-Tek, Nalge Nunc, Rochester, New York, United States) coated with gelatin. The cells were fixed with 3.7% formaldehyde for 10 min at room temperature, washed with 1× PBS, and permeabilized for at least 1 h in 70% ethanol. FISH was then performed using combinations of probes P1, P2, and P3 at a concentration of 1 ng/μl each following the procedure outlined in Femino et al. [41]. The optimal level of formamide used during hybridization and washing for maximum signal to background was empirically determined to be 25%. For the DNA FISH experiments, an additional two steps were added after the permeabilization step: (1) cells were subjected to RNase A treatment at 100 μg/ml in PBS for 30 min at 37 °C, after which (2) cells were heated to 80 °C for 8 min in 70% formamide, 2× SSC, after which the hybridization proceeded as described above.

### Image acquisition and analysis.

After in situ hybridization, cells were imaged using an Axiovert 200M inverted fluorescence microscope (Zeiss, Oberkochen, Germany), equipped with a 100× oil-immersion objective and a CoolSNAP HQ camera (Photometrics, Pleasanton, California, United States), and cooled to −30 °C; then, standard filter sets obtained from Omega Optical (Brattleboro, Vermont, United States). Openlab acquisition software (Improvision, Sheffield, United Kingdom), was used to acquire the images. For three-dimensional imaging, randomly chosen fields were imaged by taking adjacent Z-axis optical sections that were 0.3 μm apart. The particles were counted in three dimensions using custom software written in MATLAB (The Mathworks, Natick, Massachusetts, United States). The general procedure was to (i) manually select the individual cells in a field, then (ii) run a median filter on each optical slice taken, then (iii) run a custom linear three-dimensional filter, designed to enhance particulate signals and loosely based on the discrete Laplacian, on the stack of images, then (iv) manually select a threshold for the enhanced images, and then (v) count the total number of isolated signals (i.e., connected components) in three dimensions. For each manually selected threshold, other thresholds 25% above and below were also analyzed to verify that the particle count did not depend significantly on the particular threshold chosen. Our best estimate is that the number of spots counted by our algorithm is accurate to within 10% of the actual number. In cells with transcription sites, the transcription site itself was subjected to the same counting procedures, usually resulting in it being counted as a single molecule. This is justified, since the nascent RNAs present at the transcription site are mostly likely unprocessed pre-mRNA that have not yet been subjected to the various post-transcriptional modifications required for an mRNA to be considered functional [41].

In experiments where fluorophores other than TMR were used, we instead quantified the relative amount of mRNA from cell to cell by counting the number of mRNA in one optical section (chosen near the bottom of the cellular volume). This was done because the relatively poor photostability of the Cy5.5 and Alexa 594 dyes meant that the particulate signal became quite weak during the acquisition of the image stacks, making the imaging and counting of individual molecules progressively more difficult and thus significantly less accurate. In the case of the L-GFP-M1-7x clone, the large number of mRNA molecules often resulted in signals too intense to quantify using the segmentation method above due to overlap in the diffraction-limited spots. In this case, we quantified the mRNA by integrating the total fluorescence over the entire cellular volume. To relate this to the absolute number of mRNA in the cell, we quantified the mRNA in several test cells where the counting procedure was reliable using our particle counting algorithm and correlated that to the total fluorescence within the volume. The relationship was found to be linear (Figure S3), thus yielding a simple formula by which one can compute the total number of molecules in a cell given its total integrated fluorescence. While this method is most likely relatively inaccurate for low numbers of particles, it is able to yield a reasonable estimate of the number of molecules in cells with very large numbers of mRNA. The Supporting Information videos were created by deconvolving the optical sections and rendering them in three-dimensional using Volocity (Improvision).

The fluorescent protein levels were quantified by a single fluorescence image toward the lower focal plane of the cells. The total fluorescence was found by integrating the difference between the pixel intensities and the average background over the entire cellular area. In the case of the live cell YFP images, OptiMEM (Sigma) was used as growth medium because of its reduced autofluorescence as compared with regular MEM.

All software is available upon request.

### Statistical analysis and estimation of model parameters.

The error bars for the means and noises reporter were obtained by the bootstrap method. The parameters from the model were estimated using the maximum-likelihood method based on an explicit formula derived for the complete mRNA distribution as outlined in Protocol S1. The error bars reflect 95% confidence intervals.

The *p*-values for all the correlations given represent probabilities of finding the given data assuming the null hypothesis of no correlation.

The *p*-values comparing the mRNA distributions in cells either containing or not containing transcription sites and comparing the nuclear versus cytoplasmic fraction were found by a permutation method and reflect the chances of obtaining the percentages found by random chance (i.e., by randomizing which cells are labeled as transcriptional active and inactive).

### Determination of mRNA decay rate.

The mRNA decay rate was found by using real-time RT-PCR on RNA isolated from cell line L-GFP-M1-7x grown in medium containing 10 ng/ml doxycycline for a range of times using the Qiagen One-Step RT-PCR kit (Valencia, California, United States). The real time RT-PCR was performed for both the GFP transgene and the highly expressed elongation factor 1 gene, which served as an internal control not likely to change in response to doxycycline concentration. We used primers and molecular beacons specific to each gene to perform real-time PCR. The difference in threshold cycle between the GFP and the EF1 signals was linearly related to the time since transcription was halted, allowing an accurate determination of the half-life of the mRNA from the transgene. The results are shown in Figure S1. In determining the half-life, only the time points at 2, 4, and 8 h were considered so as not to confound the results with any transient behavior associated with mRNA processing and export.

### Stochastic simulations.

Stochastic simulations of the stochastic mRNA and protein model described in Protocol S1 were performed by implementing Gillespie's Direct method [30] in Matlab (The Mathworks). The parameters governing the mRNA dynamics were taken from those obtained from cell line E-YFP-M1-7x grown under conditions of no doxycycline: rate of gene activation (λ/δ) = 2.44, inactivation (γ/δ) = 2.49, and transcription (μ/δ) = 910. Since we are only interested in steady-state solutions, the mRNA degradation rate δ was chosen as a factor by which all the other rates were scaled. The translation rate μ_p_/δ was set to 100 and three values of the protein degradation rate δ_p_/δ were investigated: 0.02, 0.16, and 2.56. In Figure 7C, the values of δ and δ_p_ are reported in physical units for clarity.

## Supporting Information

Figure S1 Determination of Reporter mRNA Degradation Rate Plot shows the difference in threshold cycle between PCRs performed on the GFP reporter gene and the EF1 housekeeping from a real-time RT-PCR experiment performed on total mRNA extracted from cell line L-GFP-M1-7x. At time 0, the cellular media was replaced with media containing 10 ng/ml doxycycline, effectively shutting down transcription of the reporter gene, thus allowing for a determination of the mRNA degradation time. In determining the half-life, we only considered the rightmost three points, since early time points may display non–first-order degradation due to transient effects of mRNA processing and export.
(61 KB PDF)

Figure S2 Effects of Cellular Volume upon mRNA Noise Plot shows the noise (defined as the standard deviation divided by the mean) of mRNA concentrations for the 1x-tetO construct (red) and the 7x-tetO construct (blue) over a range of doxycycline concentrations. The mRNA concentration was determined by dividing the number of mRNA by the total volume of the cell, as determined by microscopy. Compare to Figure 3B, bottom.
(61 KB PDF)

Figure S3 Linear Correlation Used to Provide Accurate Estimates of the Number of mRNA Molecules in Heavily Expressing Cells Encountered when Analyzing mRNA Levels in the L-GFP-M1-7x Cell Line Plot shows the correlation between the number of mRNA molecules and total fluorescence integrated over the cellular volume (background subtracted). Cells were taken from random fields of cell line L-GFP-M1-7x and cells were chosen for both reasonable levels of mRNA to allow for segmentation and for lack of brightly fluorescent features that could potentially influence the calibration. The linear fit indicated a value of roughly 53,500 fluorescence units per molecule of mRNA.
(60 KB PDF)

Protocol S1 Model of Gene Activation and Inactivation, Parameter Estimation, and Comparison to Previous Studies Mechanistic model of bursts in mRNA synthesis, basic model of gene activation and inactivation, fitting of parameters to experimental distributions, determination of protein mean and variances, and relationship between model and previous studies of intrinsic versus extrinsic noise.
(106 KB PDF)

Table S1 Number of Reporter (YFP-M1) mRNAs per Cell Used in the Study from Cell Lines E-YFP-M1-1x and E-YFP-M1-7x, and Number of RNAPII Large Subunit mRNAs in Cell Line E-YFP-M1-7x (21 KB XLS)

Video S1 Three-Dimensional Flythrough of a Pair of Recently Divided Sister Cells from Cell Line E-YFP-M1-7x Each white spot represents a molecule of mRNA. The dense white spot in one of the cells is an active transcription site.
(6.0 MB MOV)

Video S2 Three-Dimensional Flythrough of a Pair of Recently Divided Sister Cells from the Cell Line Possessing Two Reporter Genes Integrated into the Same Locus (L-YFP-M1-CFP-M2) Green corresponds to the signal from YFP-M1 mRNA, and red corresponds to the signal from CFP-M2 mRNA. The dense yellow spot in both cells is an active site of transcription, indicating that both mRNAs are being transcribed at the same genomic locus.
(4.0 MB MOV)

## Abbreviations

CHO: Chinese hamster ovary
FISH: fluorescence in situ hybridization
GFP: green fluorescent protein
tTA: tet-transactivator
