# Supplementary material for: Stochastic mRNA Synthesis in Mammalian Cells
Source: PLoS Biol. 2006 Sep 12;4(10):e309. doi: 10.1371/journal.pbio.0040309 (PMC1563489; doi:10.1371/journal.pbio.0040309)
Supplement: Supplementary file 1 [file pbio.0040309.sd001.pdf]

Supplementary Information for "Stochastic mRNA Synthesis in Mammalian Cells"

Arjun Raj,[1,2] Charles S. Peskin,[1] Daniel Tranchina,[1]
Diana Y. Vargas,[2] Sanjay Tyagi[2*]

[1]Department of Mathematics, New York University,
251 Mercer St., New York, NY 10012, USA
[2]Department of Molecular Genetics
225 Warren St., Newark, NJ 07103, USA

[*]To whom correspondence should be addressed; E-mail: sanjay@phri.org.

June 13, 2006

Mechanistic model of bursts in mRNA synthesis

Here, we describe a stochastic model of gene expression which explains the mRNA bursts observed in the experiments detailed in the main text. We analyze a model of gene activation and inactivation and provide formulas for the steady-state mRNA distribution. We describe how the parameters were estimated using this formula, and also derive some results for the noise in protein levels. We end with a brief discussion of intrinsic vs. extrinsic noise in relation to the experiments upon which the model is based.

Basic model of gene activation and inactivation

In this section, we use the model of gene activation and inactivation first examined by Peccoud and Ycart [1] to find the relative rates of gene activation and inactivation from the experimental histograms in the main paper.

The model consists of a gene which transitions randomly between an active state A, in which transcription of the gene into molecules of mRNA (denoted M) is very efficient, and an inactive state I, in which transcription is not possible. In the nomenclature of the main text, the time during which the gene is in the active state A is the "burst" of mRNA synthesis. The reactions which generate the stochastic chemical master equation describing this process are as follows:

$$\begin{aligned} \mathrm{I} &\xrightarrow{\lambda} \mathrm{A} \\ \mathrm{A} &\xrightarrow{\gamma} \mathrm{I} \\ \mathrm{A} &\xrightarrow{\mu} \mathrm{A}+\mathrm{M} \\ \mathrm{M} &\xrightarrow{\delta} \emptyset \end{aligned}$$

where λ is the rate of gene activation, γ is the rate of gene inactivation, μ is the rate of transcription when the gene is in the active state, and δ is the rate of mRNA decay. We will henceforth use M to denote the number of mRNA.

A steady-state solution to the discrete master equation exists, and is given by

$$\rho(m) = \frac{\Gamma\left(\frac{\lambda}{\delta}+m\right)}{\Gamma\left(m+1\right)\Gamma\left(\frac{\lambda}{\delta}+\frac{\gamma}{\delta}+m\right)} \frac{\Gamma\left(\frac{\lambda}{\delta}+\frac{\gamma}{\delta}\right)}{\Gamma\left(\frac{\lambda}{\delta}\right)} \left(\frac{\mu}{\delta}\right)^m {}_1F_1\left(\frac{\lambda}{\delta}+m, \frac{\lambda}{\delta}+\frac{\gamma}{\delta}+m, -\frac{\mu}{\delta}\right) \quad (1)$$

where m is a non-negative integer, $\rho(m)$ represents the probability of having m mRNA, and ${}_1F_1(a,b,c)$ is a confluent hypergeometric function of the first kind (the derivation of this formula will be given in a manuscript currently in preparation).

In the limit of large γ/δ, this becomes

$$\rho(k) = \left(1+\frac{\mu}{\gamma}\right)^{-\frac{\lambda}{\delta}} \frac{\Gamma\left(\frac{\lambda}{\delta}+k\right)}{\Gamma\left(\frac{\lambda}{\delta}\right)\Gamma\left(k+1\right)} \left(\frac{\frac{\mu}{\gamma}}{1+\frac{\mu}{\gamma}}\right)^k \quad (2)$$

We used the full expression in Equation (1) for all the statistical procedures employed in this paper, as described in the "Fitting of parameters to experimental distributions" section.

An interesting parameter regime to investigate is that of μ being large compared to the other rates in the system. In this case, one can then make the approximation that M is a continuous variable. With this approximation, the dynamics can be described by

$$\frac{dM}{dt} = -\delta M + \mu f(t) \tag{3}$$

Here, $f(t) \in \{0, 1\}$ is a random telegraph signal representing the active and inactive states of the gene; the rates of switching on and off are λ and γ, respectively. To find the steady state mRNA distribution, we examine the conditional mRNA densities in both the active and inactive state. In particular, we define

$$\rho_{\mathrm{A}}(m,t)dm = \Pr\left(f(t) = 1 \text{ and } m \in (m, m+dm) \text{ at time } t\right) \tag{4}$$
$$\rho_{\mathrm{I}}(m,t)dm = \Pr\left(f(t) = 0 \text{ and } m \in (m, m+dm) \text{ at time } t\right) \tag{5}$$

The total mRNA density we are interested in, ρ, is then given by the sum of these two densities $\rho_{\mathrm{A}} + \rho_{\mathrm{I}}$. The probability flux of the two densities is determined by the rates of production and degradation:

$$\mathrm{J}_{\mathrm{A}}(m,t) = (\mu - \delta m)\rho_{\mathrm{A}}(m,t) \tag{6}$$
$$\mathrm{J}_{\mathrm{I}}(m,t) = -\delta m \rho_{\mathrm{I}}(m,t) \tag{7}$$

Using these equations for the flux, conservation of probability then yields the following equation for the time evolution of the two densities:

$$\frac{\partial \rho_{\mathrm{A}}}{\partial t} + \frac{\partial \mathrm{J}_{\mathrm{A}}}{\partial m} = \lambda \rho_{\mathrm{I}} - \gamma \rho_{\mathrm{A}} \tag{8}$$
$$\frac{\partial \rho_{\mathrm{I}}}{\partial t} + \frac{\partial \mathrm{J}_{\mathrm{I}}}{\partial m} = -\lambda \rho_{\mathrm{I}} + \gamma \rho_{\mathrm{A}} \tag{9}$$

Adding these two equations, we obtain

$$\frac{\partial}{\partial m}\left(\mu \rho_{\mathrm{A}} - \delta m \rho\right) = 0, \tag{10}$$

implying that the difference $\mu\rho_{\mathrm{A}} - \delta m \rho$ is a constant. Since the densities ρ_{A} and ρ must both go to zero as m becomes large, the constant must be zero, thus yielding the following equations expressing the conditional densities in terms of the total density:

$$\rho_{\mathrm{A}} = \frac{\delta m}{\mu}\rho \tag{11}$$
$$\rho_{\mathrm{I}} = \left(1 - \frac{\delta m}{\mu}\right)\rho \tag{12}$$
$$\tag{13}$$

Inserting these expressions into Equation (9) yields the following ordinary differential equation for ρ:

$$\frac{\partial}{\partial m}\left(-\delta m \left(1 - \frac{\delta m}{\mu}\right)\rho\right) = \left(-\lambda\left(1 - \frac{\delta m}{\mu}\right) + \gamma\frac{\delta m}{\mu}\right)\rho \tag{14}$$

whose solution gives the following expression for the steady mRNA density

$$\rho(m) = \left(\frac{\mu}{\delta}\right)^{1-\frac{\lambda}{\delta}-\frac{\gamma}{\delta}} \frac{\Gamma\left(\frac{\lambda}{\delta}+\frac{\gamma}{\delta}\right)}{\Gamma\left(\frac{\lambda}{\delta}\right)\Gamma\left(\frac{\gamma}{\delta}\right)} m^{\frac{\lambda}{\delta}-1} \left(\frac{\mu}{\delta}-m\right)^{\frac{\gamma}{\delta}-1}, \tag{15}$$

where the constant of integration is determined by the normalization condition of the density function.

As with the complete solution in Equation (1), an important limiting case is that where the rate of inactivation, γ is significantly larger than the activation rate λ and is somewhat larger than the mRNA degradation rate δ. Physically, this corresponds to the case of short but infrequent bursts of mRNA synthesis. Taking this limit, we obtain the following approximate expression for the steady state mRNA density:

$$\frac{\frac{\gamma}{\mu}}{\Gamma(\frac{\lambda}{\delta})} \left(\frac{\gamma}{\mu}m\right)^{\frac{\lambda}{\delta}-1} e^{-\frac{\gamma}{\mu}m} \tag{16}$$

This density takes the form of a Gamma distribution. In this case, the distribution is characterized by only two parameters, λ/δ and μ/γ, rather than the three parameters in the more general case. This is because, for large γ/δ, the bursts are of short duration, meaning that mRNA degradation during the burst negligible (i.e., the system rarely leaves the linear phase of the transient behavior). Since the parameter μ/γ defines the mean number of mRNA produced during the burst, the specific values of the parameters μ/δ and γ/δ are unimportant as long as their ratio is held constant.

Fitting of parameters to experimental distributions

To determine the parameters of our model, we fit our experimental data from the series of doxycycline experiments to the steady-state solution to the master equation by using the maximum likelihood method (the steady-state solution for each set of parameter values was found through by using a numerical method to quickly compute the p.d.f given by Equation (1)). For two of the cases analyzed (E-YFP-M1-7x at no doxycycline and RNA polymerase II), this resulted in well-defined values for all three parameters, μ/δ, λ/δ, and γ/δ. However, when examining the log-likelihood keeping λ/δ fixed while varying γ/δ and μ/δ, we found that in the other cases, the maximum likelihood was spread along a "ridge" corresponding to a constant ratio of γ/μ. This indicated that there was insufficient experimental data to resolve the parameters γ/δ and μ/δ. This is most likely because the parameters fell in the regime of large γ/δ, which, as outlined above, results in a gamma distribution in which only the ratio of γ to μ is important. Rather than using the approximate gamma distribution to find the value of μ/γ from the experimental data, we instead fitted the the data to the full model while holding μ/δ fixed, because letting μ/δ vary resulted in unphysically large values of μ/δ. The values used for μ/δ were 500 for cell line E-YFP-M1-1x and 910 for cell line E-YFP-M1-7x (in the former case, a physically reasonable value was chosen, whereas in the latter case, the value of μ/δ was chosen to be that obtained in the case of E-YFP-M1-7x grown with no doxycycline, in which the value could be determined from the distribution itself). Varying these values by 25% typically did not change the parameters λ/δ and μ/γ by

more than 10% at most. 95% confidence intervals for the parameters were found by varying one parameter while holding the others fixed and finding the value for the parameter that decreased the likelihood by a factor of 0.05.

To show that the theoretical distributions generated by the parameters estimated by the maximum likelihood method actually matched our experimental ones, we generated a large set of likelihood values for data of the same size as our experimental sample (generated from the model by a Monte Carlo method). We found that in all cases, the data was in the middle 50th percentile, well within the middle 90th percentile that would indicate that the experimental data was consistent with the model used.

Determination of protein mean and variances

In this section, we compute the mean and the variance of the protein distribution. These expressions can yield insight into the key parameters contributing to cellular noise.

To begin, we use the approximation that μ is large, thereby allowing us to use Equation (3) to describe the mRNA dynamics. The mean mRNA level of this system is given by

$$\langle M \rangle = \frac{\mu}{\delta} \frac{\lambda}{\lambda + \gamma} \tag{17}$$

To find the variance of the mRNA level in this system over time, we first subtract the mean to produce a mean-zero process, yielding

$$\frac{d\tilde{M}}{dt} = -\delta \tilde{M} + \mu \tilde{f}(t)$$

This system has an impulse response

$$h(t) = H(t)\mu e^{-\delta t}$$

where H(t) is the Heaviside function. Now let $R_{\tilde{M}\tilde{M}}(\tau)$ be the autocorrelation function of $\tilde{M}$. Upon taking the Fourier transform, we obtain

$$\hat{R}_{\tilde{M}\tilde{M}}(\omega) = \left|\hat{h}(\omega)\right|^2 \hat{R}_{\tilde{f}\tilde{f}}(\omega)$$

Inverting the Fourier transform yields

$$R_{\tilde{M}\tilde{M}}(0) = \frac{1}{2\pi} \int_{-\infty}^{\infty} \left|\hat{h}(\omega)\right|^2 \hat{R}_{\tilde{f}\tilde{f}}(\omega) d\omega$$

where $R_{\tilde{M}\tilde{M}}(0) = \left\langle \tilde{M}^2 \right\rangle = \text{var}(M)$, which is the desired quantity. The autocorrelation of f is given by

$$R_{\tilde{f}\tilde{f}}(\tau) = \frac{\lambda\gamma}{(\lambda + \gamma)^2} e^{-(\lambda+\gamma)|\tau|}$$

Taking the Fourier transform and evaluating the integral gives the desired formula for the variance:

$$\text{var}(M) = R_{\tilde{M}\tilde{M}}(0) = \frac{\mu^2 \lambda\gamma}{\delta(\gamma + \lambda)^2(\delta + \gamma + \lambda)} \tag{18}$$

This is the same as is obtained by taking the limit of large μ in the result computed by Peccoud and Ycart [1] using moment generating functions to find the variance directly from the master equation. However, our derivation shows that the result is still valid even if the only stochastic element of the system is the telegraph forcing term corresponding to gene activation, indicating that the random nature of the individual events of mRNA synthesis and degradation are not particularly important to the overall stochastic behavior of the system.

Using the expressions for the mean and variance obtained above, one finds the following expression for the square of the noise, where we define the noise η as the standard deviation divided by the mean:

$$\eta^2 = \frac{1}{\lambda}\left(\frac{\delta\gamma}{\delta+\lambda+\gamma}\right) \tag{19}$$

To model the protein dynamics, we extend the model of mRNA dynamics presented above. The set of chemical reactions now include the production and degradation of the proteins p:

$$\begin{aligned}
\mathrm{I} &\overset{\lambda}{\to} \mathrm{A} \\
\mathrm{A} &\overset{\gamma}{\to} \mathrm{I} \\
\mathrm{A} &\overset{\mu}{\to} \mathrm{A+M} \\
\mathrm{M} &\overset{\delta}{\to} \emptyset \\
\mathrm{M} &\overset{\mu_p}{\to} \mathrm{M+P} \\
\mathrm{P} &\overset{\delta_p}{\to} \emptyset
\end{aligned}$$

As before, λ and γ are the gene activation and inactivation rates, respectively, and μ and δ are the mRNA synthesis and decay rates. μ_p is the protein synthesis (i.e., translation) rate (per mRNA molecule) and δ_p is the rate of protein degradation, and we use P to refer to the number of protein molecules P. Equation (3) gives a differential equation for the dynamics of the mRNA which is driven by a random telegraph signal f representing the gene activation and inactivation events. The corresponding equation for the protein dynamics is

$$\frac{dP}{dt} = -\delta_p P + \mu_p M(t) \tag{20}$$

where M is the number of mRNA molecules. The mean number of proteins is given by

$$\langle P\rangle = \frac{\mu_p\,(\mu/\delta)}{\delta_p}\frac{\lambda}{\lambda+\gamma} \tag{21}$$

obtained by simply multiplying the mean number of mRNA molecules in Equation (17) by μ_p/δ_p.

To find the variance of the protein levels over time, we again turn the system into a mean-zero process by subtracting the mean, yielding

$$\frac{d\tilde{P}}{dt} = -\delta_p \tilde{P} + \mu_p \tilde{M}(t) \tag{22}$$

where $\tilde{P} = P - \langle P \rangle$. The impulse response of this system is now given by

$$h_p(t) = H(t)\mu_p e^{-\delta_p t}$$

where $H(t)$ is the Heaviside function.

As before, we find the Fourier transform of the autocorrelation function of $\tilde{P}$, denoted $R_{\tilde{P}\tilde{P}}$, is given by

$$\hat{R}_{\tilde{P}\tilde{P}}(\omega) = \left|\hat{h}_p(\omega)\right|^2 \left|\hat{h}(\omega)\right|^2 \hat{R}_{\tilde{f}\tilde{f}}(\omega) \tag{23}$$

Again, one can obtain the variance by integrating, whence

$$\mathrm{var}(P) = \frac{\mu^2\mu_p^2(\gamma\lambda)(\delta+\delta_p+\lambda+\gamma)}{(\lambda+\gamma)^2\delta\delta_p(\delta+\delta_p)(\delta+\lambda+\gamma)(\delta_p+\lambda+\gamma)} \tag{24}$$

The square of the protein noise is then given by

$$\eta_p^2 = \frac{\gamma}{\lambda}\frac{\delta\delta_p}{\delta+\delta_p}\frac{\delta+\delta_p+\lambda+\gamma}{(\delta+\lambda+\gamma)(\delta_p+\lambda+\gamma)} \tag{25}$$

One important case to consider is that of fast mRNA degradation, i.e., $\delta \gg \delta_p$. This assumption results in the following simplified expression for the protein noise:

$$\eta_p^2 = \frac{1}{\lambda}\frac{\delta_p\gamma}{\delta_p+\lambda+\gamma} \tag{26}$$

Notice that this equation is virtually identical to Equation (19), except that δ is replaced by δ_p. This makes intuitive sense: if the protein decay rate is very small compared to the mRNA decay rate, then the activation state of the gene is essentially reflected in the mRNA level. On the time scale of the proteins, it will essentially see a telegraph-like mRNA signal, thus resulting in identical noise properties to that of the mRNA. This is an important simplification and we will adopt it for our analysis in many of the following sections.

Interestingly, a small protein decay rate can significantly reduce fluctuations in protein levels even when genes only switch into a *transcriptionally* active state from time to time; i.e., when λ is small. Physically, this situation is one where the proteins are generally quite abundant, and the occasional mRNA burst merely serves to make up for the proteins lost due to slow protein decay. In this case, the distribution is very nearly Gaussian, and so the expression for the second order moments are actually physically meaningful. One can see this in the protein probability density functions shown in Figure 7C.

Relationship between model and previous studies of intrinsic vs. extrinsic noise

The fact that the total noise (the combination of the contributions from both extrinsic and intrinsic noise sources) observed in the E-YFP-M1-7x and L-GFP-M1-7x cell lines varies non-monotonically as the overall level of gene expression is decreased is similar to observations of the dependence of *total* noise upon the overall level of gene expression found in previous

studies [2, 3, 4]. As mentioned in the main text, if the noise were due to mRNA dynamics being governed by a simple birth-death process, one would expect that a monotonic decrease in the mean would result in a monotonic increase in the noise: in that case, both the mean and variance of the number of mRNA per cell would be μ/δ, where μ is the rate of mRNA transcription and δ is the rate of mRNA decay. Thus, since the standard deviation is the square root of the variance, a doubling of the mean would result in a reduction of the variance by $\sqrt{2}$. This characteristic "square root" decrease in noise is often observed in stochastic processes and is often referred to as the central limit theorem.

In the two studies where the authors were able to separate the total noise into its extrinsic and intrinsic components [2, 4], the authors found that while the extrinsic noise also displayed the characteristic non-monotonicity, the intrinsic noise decreased monotonically as the level of gene expression increased, as is consistent with a stochastic process. This lead Raser and O'Shea [4] to conclude that the non-monotonicity in the total noise observed by Blake et al. [3] was primarily due to extrinsic noise.

In our experiments, we are unable to quantitatively determine the relative contributions of extrinsic and intrinsic noise to the total noise, since doing so requires that the two reporters used are statistically identical [2, 5]. This is impossible in the case of mammalian cells, since each cell line established displays different behaviors depending on where the gene was randomly integrated. However, our experiments where the two reporter genes are integrated into different genomic loci shows that there is no correlation at all between the two reporters used (Fig. 5B). Qualitatively, this shows that the extrinsic contribution to the total noise is very low; any significant extrinsic contribution would manifest itself as a detectable correlation. This validates one of the key implicit assumptions of the model presented, namely that one may safely neglect extrinsic factors.

There are two other corroborating pieces of evidence that the non-monotonic noise profile displayed in the E-YFP-M1-7x and L-GFP-M1-7x cell lines is not due to an inability to distinguish between intrinsic and extrinsic noise. First, the noise in the E-YFP-M1-1x cell line did not display this non-monotonicity; were the non-monotonic profile due to some general mechanism of extrinsic noise generation, one would expect it to affect this cell line as well. Second, Elowitz et al. [2] traced the source of the extrinsic noise back to variations in a transcriptional activator shared between the two genes. They showed [5] that titrations in the variations of this activator as the level of induction was altered could result in a non-monotonic noise profile. However, that possibility is explicitly ruled out here, as we have shown that variations in the level of the transcriptional activator tTA have no bearing on the noise observed.

Our finding that the mRNA noise in mammalian cells is primarily intrinsic is at odds with the results from previous studies performed with fluorescent protein reporters indicating that the noise in yeast was primarily extrinsic rather than intrinsic [4, 6]. The correlations they observed may be partly due to global variations in the rates of *protein* translation and decay, which are extrinsic factors excluded in our experiments. We should point out, though, that the mRNA and protein levels in the L-GFP-M1-7x cell line correlate rather well (Figure 7A). This may, however, be due to the fact that the GFP expressed was a destabilized variant with a half-life on the order of the mRNA itself. It is also possible that the use of fluorescence assisted cell sorting (FACS) in those studies could introduce confounding effects such as cell-size that might be counted as extrinsic variations. Some new evidence indicates that this

may be the case, as the specifics of the gatings used in the analysis of FACS data can change the measurements of the relative contributions of intrinsic and extrinsic variations [7]. An alternate explanation may lie in the work of Becskei et al. [8], where the authors show that there is correlation in gene activity between homologous loci; this may also help explain the findings of Raser and O'Shea [4].

References

[1] J. Peccoud and B. Ycart. Markovian modeling of gene-product synthesis. *Theoretical Population Biology*, 48(2):222–234, 1995. Times Cited: 18 Article English Cited References Count: 15 Rz413.

[2] M. B. Elowitz, A. J. Levine, E. D. Siggia, and P. S. Swain. Stochastic gene expression in a single cell. *Science*, 297(5584):1183–6, 2002. 1095-9203 Journal Article.

[3] W. J. Blake, K. AErn M, C. R. Cantor, and J. J. Collins. Noise in eukaryotic gene expression. *Nature*, 422(6932):633–7, 2003. 0028-0836 Journal Article.

[4] J. M. Raser and E. K. O'Shea. Control of stochasticity in eukaryotic gene expression. *Science*, 304(5678):1811–4, 2004. 1095-9203 Journal Article.

[5] P. S. Swain, M. B. Elowitz, and E. D. Siggia. Intrinsic and extrinsic contributions to stochasticity in gene expression. *Proc Natl Acad Sci U S A*, 99(20):12795–800, 2002. 0027-8424 Journal Article.

[6] D. Volfson, J. Marciniak, W.J. Blake, N. Ostroff, L.S. Tsimring, and J. Hasty. Origins of extrinsic variability in eukaryotic gene expression. *Nature*, 439(7078):861–4, 2006.

[7] J.R. Newman, S. Ghaemmaghami, J. Ihmels, D.K. Breslow, M. Noble, J.L. Derisi, and J.S. Weissman. Single-cell proteomic analysis of s. cerevisiae reveals the architecture of biological noise. *Nature*, Advanced online publication, 2006.

[8] A. Becskei, B. B. Kaufmann, and A. van Oudenaarden. Contributions of low molecule number and chromosomal positioning to stochastic gene expression. *Nat Genet*, 37(9):937–44, 2005. 1061-4036 Journal Article.
