# Supplementary material for: Stochastic mRNA Synthesis in Mammalian Cells
Source: PLoS Biol. 2006 Sep 12;4(10):e309. doi: 10.1371/journal.pbio.0040309 (PMC1563489; doi:10.1371/journal.pbio.0040309)
Supplement: Supplementary file 2 [file pbio.0040309.sg001.pdf]

Figure S1:

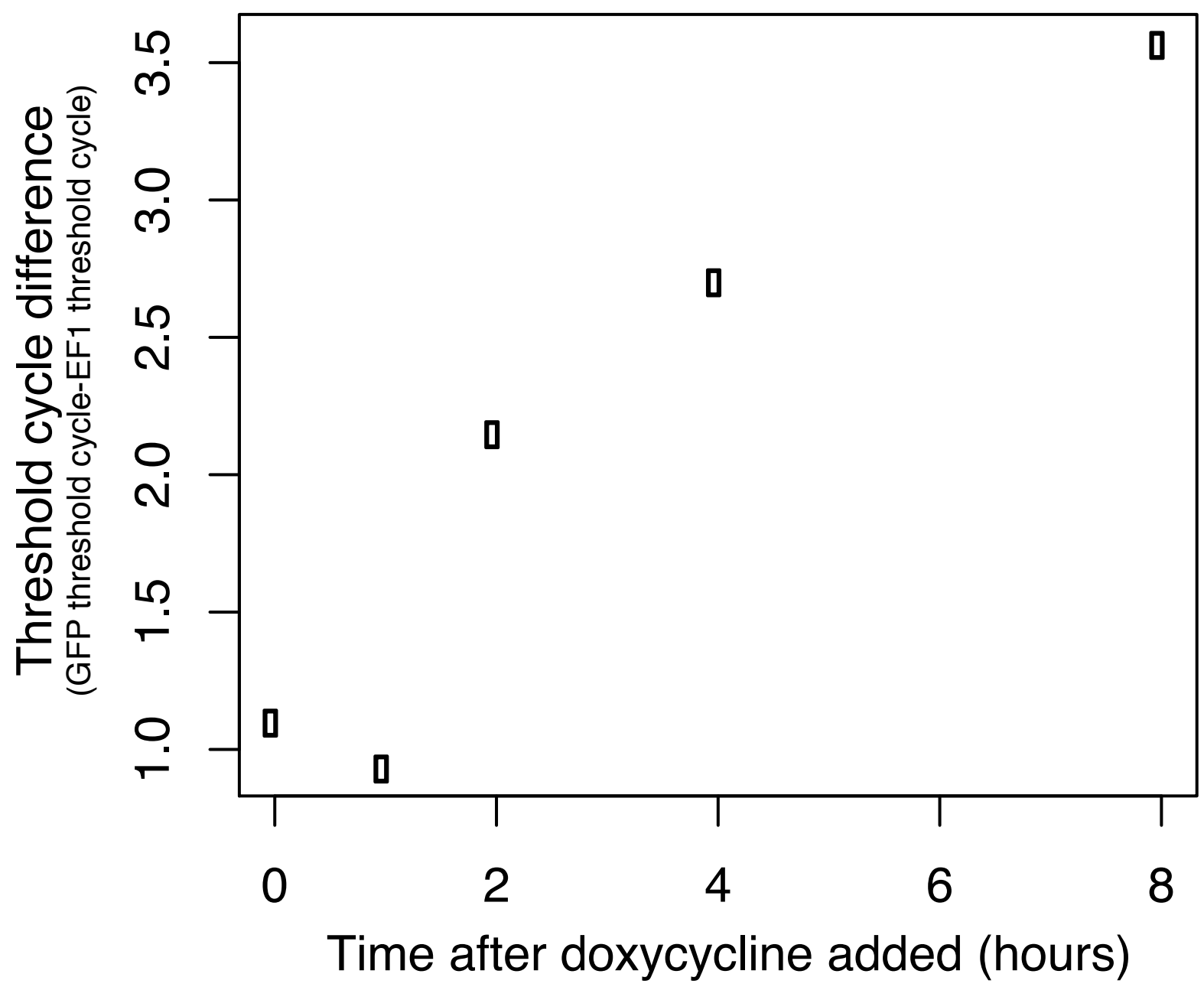

Fig. S1. Determination of reporter mRNA degradation rate. Plot shows the difference in threshold cycle between PCRs performed on the GFP reporter gene and the EF1 housekeeping from a real-time RT-PCR experiment performed on total mRNA extracted from cell line L-GFP-M1-7x. At time 0, the cellular media was replaced with media containing 10 ng/ml of doxycycline, effectively shutting down transcription of the reporter gene, thus allowing for a determination of the mRNA degradation time. In determining the half-life, we only considered the rightmost three points, since early time points may display non-first-order degradation due to transient effects of mRNA processing and export.
