# Supplementary material for: Stochastic mRNA Synthesis in Mammalian Cells
Source: PLoS Biol. 2006 Sep 12;4(10):e309. doi: 10.1371/journal.pbio.0040309 (PMC1563489; doi:10.1371/journal.pbio.0040309)
Supplement: Supplementary file 3 [file pbio.0040309.sg002.pdf]

Figure S2:

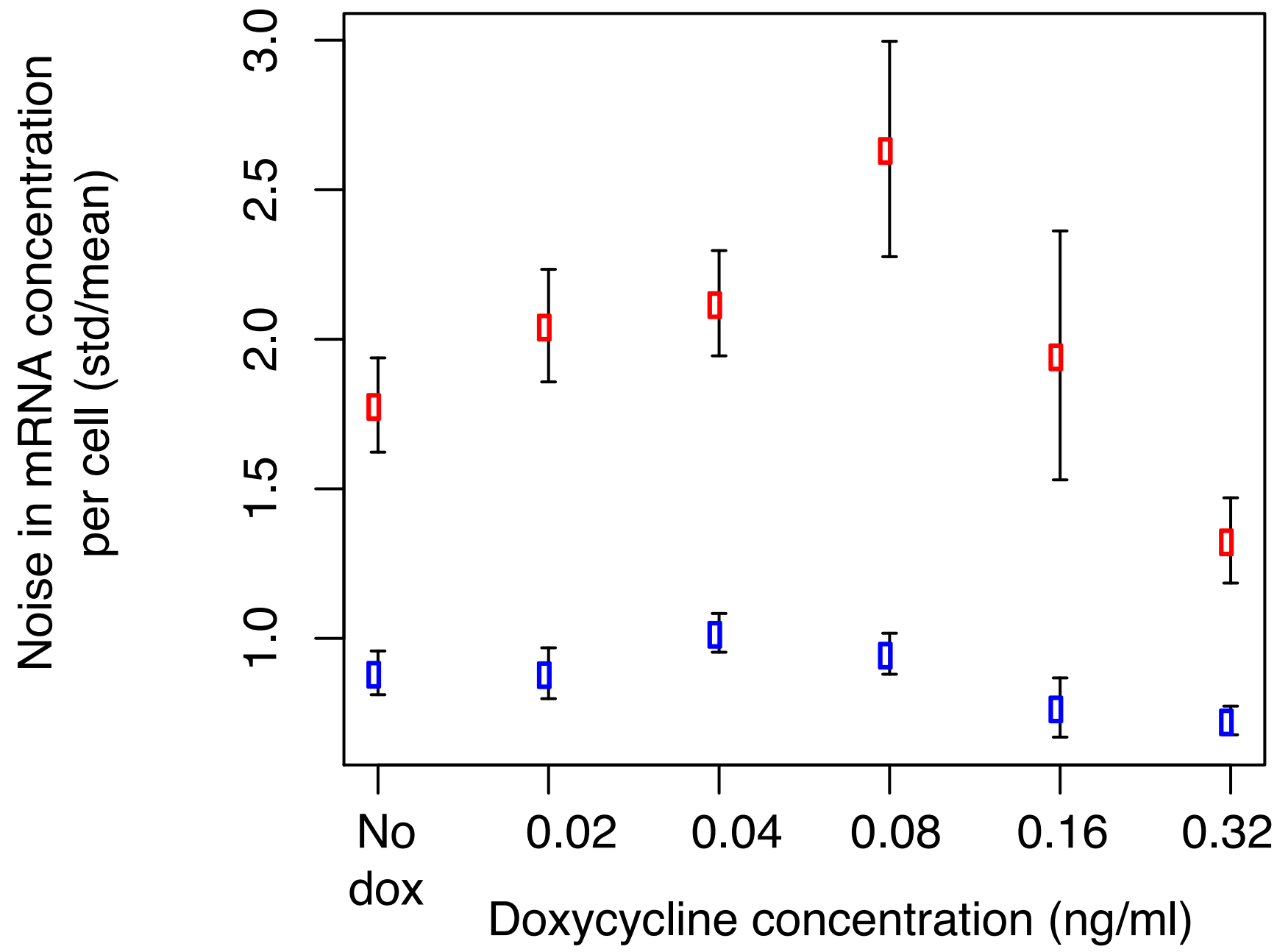

Fig. S2. Effects of cellular volume upon mRNA noise. Plot shows the noise (defined as the standard deviation divided by the mean) of mRNA concentrations for the 1x-tetO construct (red) and the 7x-tetO construct (blue) over a range of doxycycline concentrations. The mRNA concentration was determined by dividing the number of mRNA by the total volume of the cell, as determined by microscopy. Compare to Figure 3B, bottom panel.
