# Supplementary material for: Stochastic mRNA Synthesis in Mammalian Cells
Source: PLoS Biol. 2006 Sep 12;4(10):e309. doi: 10.1371/journal.pbio.0040309 (PMC1563489; doi:10.1371/journal.pbio.0040309)
Supplement: Supplementary file 4 [file pbio.0040309.sg003.pdf]

Figure S3:

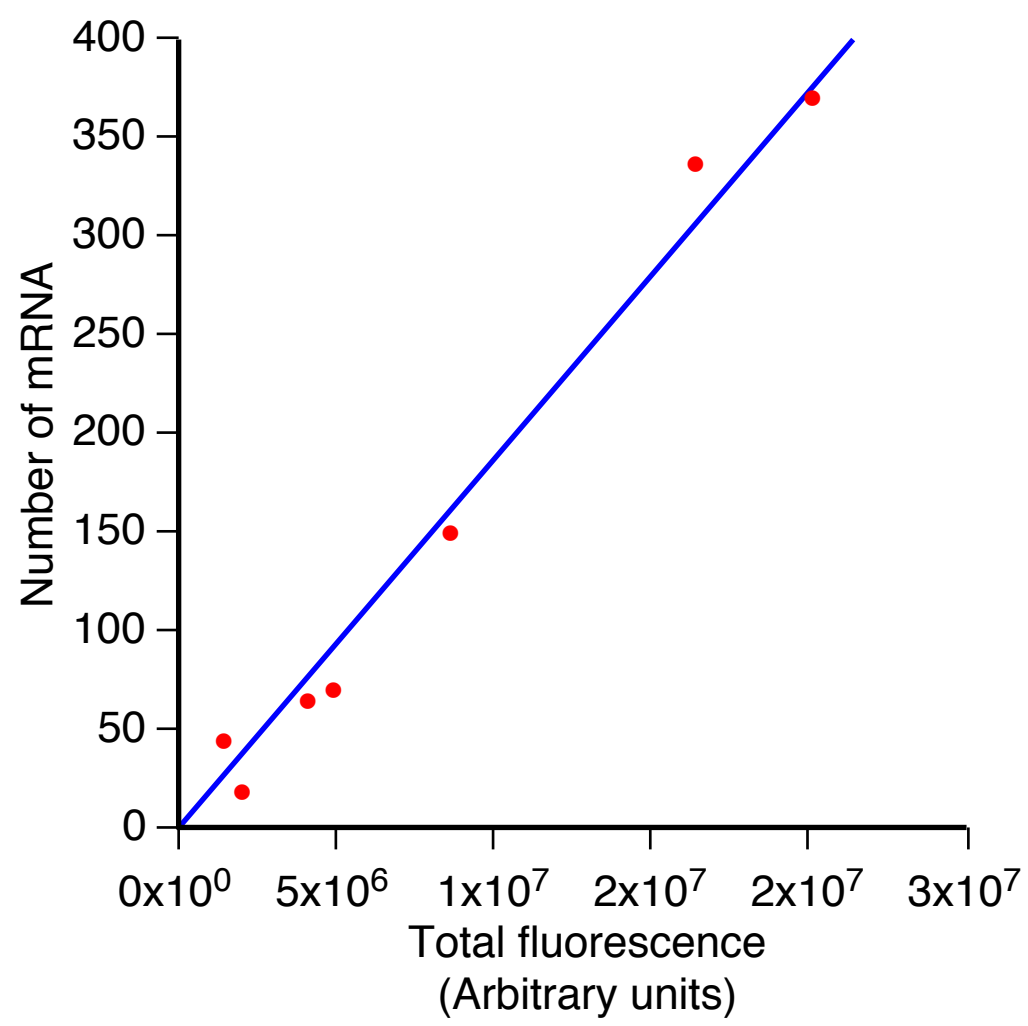

Fig. S3. Linear correlation used to provide accurate estimates of the number of mRNA molecules in heavily expressing cells encountered when analyzing mRNA levels in the L-GFP-M1-7x cell line. Plot shows the correlation between the number of mRNA molecules and total fluorescence integrated over the cellular volume (background subtracted). Cells were taken from random fields of cell line L-GFP-M1-7x and cells were chosen for both reasonable levels of mRNA to allow for segmentation and for lack of brightly fluorescent features that could potentially influence the calibration. The linear fit indicated a value of roughly 53,500 fluorescence units per mRNA.
